# AFBF-YOLO: An Improved YOLO11n Algorithm for Detecting Bunch and Maturity of Cherry Tomatoes in Greenhouse Environments

**DOI:** 10.3390/plants14162587

**Published:** 2025-08-20

**Authors:** Bo-Jin Chen, Jun-Yan Bu, Jun-Lin Xia, Ming-Xuan Li, Wen-Hao Su

**Affiliations:** College of Engineering, China Agricultural University, 17 Qinghua East Road, Haidian, Beijing 100083, China; 2022307150816@cau.edu.cn (B.-J.C.); bujunyan1@gmail.com (J.-Y.B.); 2022307150817@cau.edu.cn (J.-L.X.); 2022307150727@cau.edu.cn (M.-X.L.)

**Keywords:** precision agriculture, YOLO11, cherry tomato, ripeness detection, greenhouse environment

## Abstract

Accurate detection of cherry tomato clusters and their ripeness stages is critical for the development of intelligent harvesting systems in modern agriculture. In response to the challenges posed by occlusion, overlapping clusters, and subtle ripeness variations under complex greenhouse environments, an improved YOLO11-based deep convolutional neural network detection model, called AFBF-YOLO, is proposed in this paper. First, a dataset comprising 486 RGB images and over 150,000 annotated instances was constructed and augmented, covering four ripeness stages and fruit clusters. Then, based on YOLO11, the ACmix attention mechanism was incorporated to strengthen feature representation under occluded and cluttered conditions. Additionally, a novel neck structure, FreqFusion-BiFPN, was designed to improve multi-scale feature fusion through frequency-aware filtering. Finally, a refined loss function, Inner-Focaler-IoU, was applied to enhance bounding box localization by emphasizing inner-region overlap and focusing on difficult samples. Experimental results show that AFBF-YOLO achieves a precision of 81.2%, a recall of 81.3%, and an mAP@0.5 of 85.6%, outperforming multiple mainstream YOLO series. High accuracy across ripeness stages and low computational complexity indicate it excels in simultaneous detection of cherry tomato fruit bunches and fruit maturity, supporting automated maturity assessment and robotic harvesting in precision agriculture.

## 1. Introduction

Cherry tomatoes are a widely cultivated horticultural crop valued for their nutritional richness, vivid coloration, and strong commercial appeal [1,2]. Due to their small size, delicate texture, and clustered growth pattern, cherry tomatoes are highly susceptible to mechanical damage, necessitating careful handling during harvesting [3,4]. Traditionally, harvesting has depended heavily on manual labor, which is time-intensive, inefficient, and subject to inconsistency due to subjective ripeness assessment [5]. With the increasing automation of modern agriculture [6], accurate detection of cherry tomato clusters and precise assessment of maturity stages have become crucial for the advancement of intelligent harvesting systems [7,8].

However, detecting cherry tomatoes in complex greenhouse environments remains challenging. Challenges include leaf occlusion, overlapping clusters, inconsistent lighting, and subtle differences in ripeness stages [9]. Additionally, dynamic growth conditions and structural variability among tomato cultivars further hinder effective feature extraction [10]. These factors degrade the performance of traditional object detection algorithms, which often struggle to maintain both accuracy and robustness in real-time agricultural scenarios.

To address these challenges, early research employed image processing techniques such as color segmentation, morphological operations, and conventional machine learning algorithms to identify and localize fruits [3,11,12]. In recent years, deep learning models have shown superior accuracy and robustness in real-world agricultural environments, owing to their strong feature representation capabilities. Tenorio et al. [13] employed convolutional neural networks (CNNs) to detect and count tomato clusters, and used regression models to estimate fruit ripeness. Dai et al. [14] introduced a method that combines YOLOv5 with depth information fusion to accurately detect and track both tomato clusters and individual fruits, effectively reducing background interference. Rong et al. [15] proposed YOLOv5-4D, a model that fuses RGB and depth images, achieving a detection accuracy of 97.9%. When combined with the ByteTrack algorithm, the system was capable of stable fruit cluster counting at 40 FPS under complex backgrounds. Li et al. [16] proposed a multi-task perception network, MTA-YOLACT, capable of simultaneously detecting fruit clusters and segmenting fruit stems and main stems. Additionally, a classification and regression tree (CART) model was constructed based on spatial relationships among segmented components, enabling rapid and accurate classification of complete tomato clusters.

Although tomato cluster detection and counting have advanced significantly, most existing methods still struggle to accurately assess fruit ripeness under real-world production conditions. As modern agriculture moves toward stage- and grade-based harvesting, vision systems capable of simultaneously locating fruits and evaluating ripeness are essential to improving the practicality and intelligence of automated harvesting. Li et al. [17] proposed a multi-task YOLOv5-based model that detects both tomato clusters and individual fruits while estimating detailed ripeness levels, thereby supporting more refined harvesting strategies. Recent studies have increasingly adopted multi-task learning frameworks, enabling a single model to predict cluster locations, fruit counts, and ripeness levels [18]—thus providing both theoretical and technical support for the present study. Despite the notable progress achieved with YOLOv5 and its variants, there is limited evidence of YOLOv11-based models being applied in the domain of agricultural fruit detection, particularly for cherry tomatoes. YOLOv11, as a more recent advancement in the YOLO family, offers enhanced feature extraction capabilities and computational efficiency. However, its potential remains largely unexplored in the context of real-time, multi-task fruit perception under occlusion and ripeness ambiguity. To the best of our knowledge, this study is the first to extend YOLOv11n for the dual tasks of cluster detection and ripeness classification in dense horticultural environments. This novelty not only fills a gap in current literature but also sets a new benchmark for high-precision and lightweight detection frameworks in smart agriculture.

Moreover, existing methods remain limited in their effectiveness when applied to small-sized fruits such as cherry tomatoes. In particular, during early growth stages—when fruits have not yet changed color and exhibit low contrast with the background—the green color of the fruit closely resembles surrounding foliage, often leading to missed detections or incorrect ripeness classification. This significantly compromises the system’s generalization and stability in complex environments. To address these challenges, a multi-task visual perception framework has been developed for cherry tomato harvesting, capable of performing cluster detection and ripeness classification concurrently. Experimental results demonstrate that the proposed model achieves high detection accuracy across all ripeness stages. Notably, even in early growth stages—when fruits lack color differentiation and exhibit low contrast with the background—the model maintains high detection and classification accuracy, thereby enhancing system reliability and practical applicability in complex greenhouse environments.

To overcome the aforementioned limitations and advance the practical deployment of intelligent harvesting systems, an enhanced object detection network based on YOLO11n, named AFBF-YOLO, is proposed. To the best of our knowledge, AFBF-YOLO is the first network specifically designed to tackle dense, occluded, and ripeness-sensitive cherry tomato scenarios using a multi-task learning approach. AFBF-YOLO introduces three key innovations: (1) ACmix [19], a hybrid attention module that improves detection under occlusion by integrating convolutional and self-attention features; (2) FreqFusion [20]-BiFPN [21], a novel neck structure that captures multi-scale features and enhances boundary recognition through frequency-aware processing; and (3) Inner [22]-Focaler [23]-IoU, a refined loss function that emphasizes precise localization in dense clusters with small and overlapping targets. The primary contributions of this article are as follows:

- A high-quality cherry tomato dataset was constructed, labeled into four maturity stages and cluster categories. Data augmentation techniques were applied to simulate various environmental conditions.
- The AFBF-YOLO network was developed by integrating ACmix, FreqFusion-BiFPN, and Inner-Focaler-IoU, aiming to enhance detection accuracy and robustness under challenging scenarios.
- Extensive experiments and ablation studies were carried out, demonstrating that AFBF-YOLO significantly outperforms mainstream YOLO networks in both detection precision and computational efficiency.
- A practical and scalable solution was established for the simultaneous detection of cherry tomato clusters and their maturity stages in real time, providing a foundational step toward the integration of AI-based perception systems into robotic harvesting platforms.

The remainder of this paper is structured as follows: Section 2 describes the creation of the dataset, model improvements, and evaluation metrics. Section 3 provides a detailed account of the various experiments. Section 4 provides the discussion and an outlook for future work. Finally, Section 5 summarizes the research findings.

## 2. Materials and Methods

### 2.1. Data Collection and Dataset Construction

#### 2.1.1. Data Sample Collection

**Figure 1:**
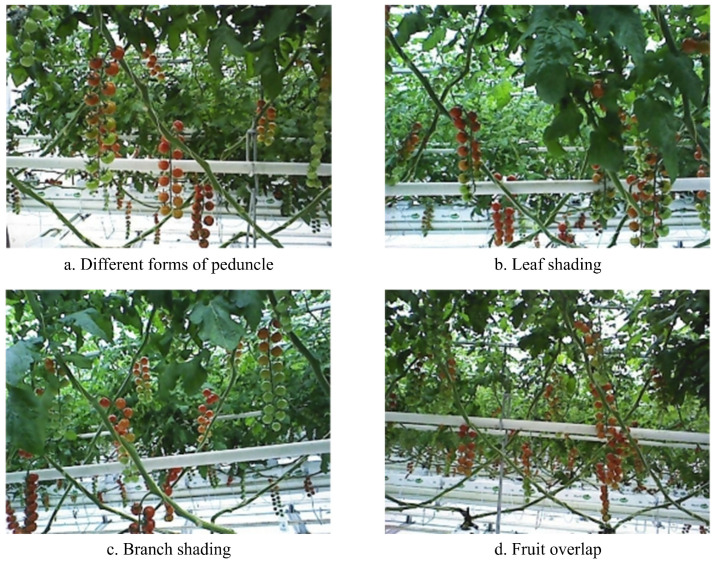
Examples image of cherry tomato dataset. (**a**) Different forms of peduncle. (**b**) Leaf shading. (**c**) Branch shading. (**d**) Fruit overlap.

In this study, images of cherry tomatoes were captured using an Astra S RGB-D camera, which records RGB images at a resolution of 640 × 480 pixels. We collected 486 RGB images of cherry tomatoes (variety Vitalin) on an experimental smart greenhouse platform (Beijing Agricultural Vocational College, Beijing, China) on 12 December 2024. The longitude and latitude of the location are 116.221292° E and 39.77127° N, respectively. Each image contained multiple fruit clusters, with individual clusters comprising numerous fruits. Fruit geometry variations caused by gravitational effects correlate with peduncle rigidity and fruit count per cluster. The greenhouse was planted and managed according to Dutch standards. An operator acquired images at 400–600 mm from the targets using a hand-held camera. To ensure diversity in the samples, these images included multiple growth stages. Thus, the dataset includes different growth stages and various orientations of tomato clusters. As illustrated in Figure 1, the collected dataset effectively reflects the real-world spatial distribution of cherry tomatoes in the greenhouse, while the data were collected on a single day at a specific location, several measures were taken to enhance the model’s generalization ability. First, the images encompass various growth stages and orientations, capturing a wide range of morphological and visual variations. Second, environmental variability was simulated through extensive data augmentation techniques during training, including changes in brightness, contrast, and color space, to approximate seasonal and lighting differences. Moreover, although the dataset is based on the Vitalin cultivar, the proposed model focuses on feature-level learning of shape, texture, and ripeness cues, which are partially shared across tomato varieties. Future work will expand the dataset to include multiple cultivars, seasons, and greenhouses to further validate cross-domain performance.

#### 2.1.2. Dataset Processing and Annotation

**Figure 2:**
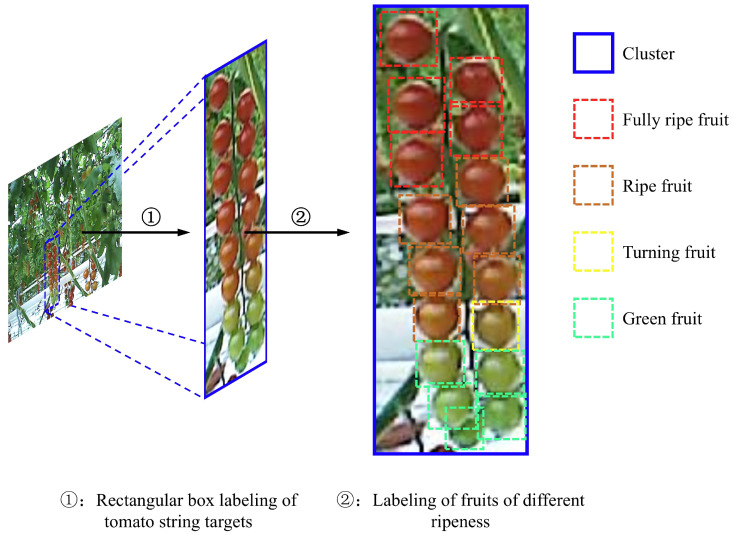
Cherry tomato dataset labeling illustration.

Considering the structure of the robotic arm and the growth habits of cherry tomatoes, only the fruits located on the front side of the cultivation shelf were selected as valid picking targets. Therefore, tomatoes on the rear side or on other cultivation shelves were not labeled. The collected images were manually annotated using the LabelMe tool, with rectangular bounding boxes applied to the targets. During labeling, each bounding box was drawn to include the entire tomato bunch along with its peduncle. After annotation, the category and bounding box location data were saved in XML format. According to agronomic standards, targets were categorized into five types: tomato bunches and fruits at four maturity stages [24]—(1) green: fully expanded with whitish-green skin, (2) turning: partial color change from white to red at the top, (3) ripe: more than 75% red or yellow coloration, and (4) fully ripe: completely red skin. The different target types and their corresponding bounding box colors are illustrated in Figure 2. The dataset was split into a training set (388 images) and a test set (98 images) at an 8:2 ratio. The training set was used to train the model, while the test set was used for evaluation. It is worth noting that only front-facing tomatoes were labeled in this dataset, as the robotic harvesting system currently operates based on a front-view perception paradigm, with picking arms approaching from the cultivation shelf’s frontal plane. This design reflects practical constraints in real-world greenhouse harvesting scenarios, where rear-facing fruits are often inaccessible or occluded. As a result, training the model on front-facing targets aligns with actual deployment requirements and optimizes task-relevant performance. However, we acknowledge that this may limit the model’s ability to detect tomatoes from oblique or rear angles. To address this in future work, we plan to collect and annotate multi-angle views—including side and oblique perspectives—by integrating mobile robot platforms and multi-camera setups, thereby enabling broader perception capabilities for more flexible harvesting strategies.

#### 2.1.3. Data Augmentation

**Figure 3:**
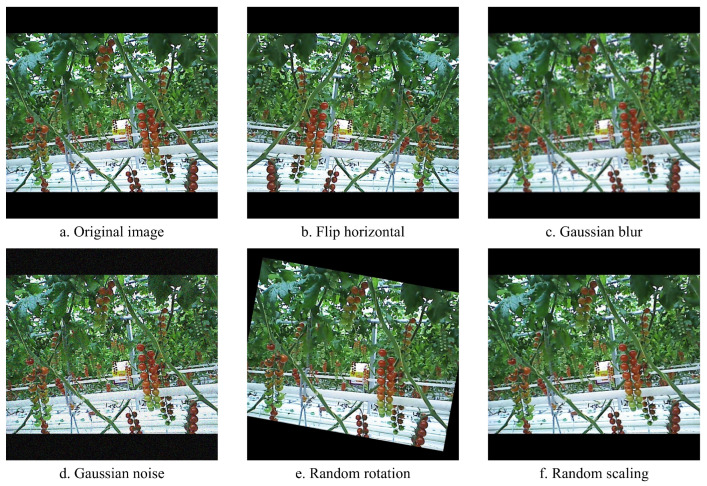
Effects of data augmentation. (**a**) Original image. (**b**) Flip horizontal. (**c**) Gaussian blur. (**d**) Gaussian noise. (**e**) Random rotation. (**f**) Random scaling.

To improve the model’s recognition performance in complex environments and mitigate overfitting caused by limited training samples, offline data augmentation was applied to the original images prior to training [25]. The original images, with a resolution of 640 × 480 pixels, were resized to 640 × 640 by adding black padding to meet the input size requirements of the model. Subsequently, the 486 images were divided into a training set and test set using an 8:2 ratio. Various augmentation techniques were implemented on the training set using Python scripts, including (1) horizontal flipping with remapping of target center coordinates to maintain annotation accuracy; (2) Gaussian blur to improve robustness against image sharpness variations; (3) addition of Gaussian noise to simulate real-world sensor noise; (4) random rotation to enhance robustness against changes in object orientation; and (5) random scaling to improve detection across varying object sizes. As shown in Figure 3, each original image generated five new images. These augmentation strategies increased the number of training images to 2328, while the test set remained at 98 images, thereby enriching the dataset and enhancing the model’s robustness and generalization ability. The final distribution of each target category is summarized in Table 1.

### 2.2. YOLO11 Model

The YOLO series has become one of the most widely used real-time object detection algorithms, and YOLO11, developed by the Ultralytics team, is the latest version offering improved speed and accuracy compared to its predecessors [26]. The YOLO11 framework includes five model variants: n, s, m, l, and x. The lightweight version, YOLO11n, was selected as the base model in this study to reduce computational and storage requirements, thereby facilitating deployment on edge devices for integration into harvesting robots.

**Figure 4:**
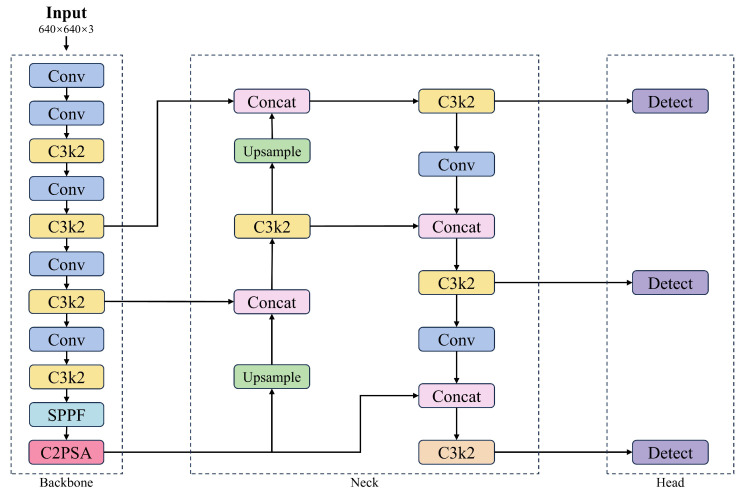
The network structure of YOLO11.

The YOLO11 architecture comprises a backbone, neck, and head. Compared to YOLOv8 [27], YOLO11 replaces the Cf2 module with C3k2 and introduces a C2PSA module after the SPPF component. Additionally, YOLO11 adopts the head design from YOLOv10 [28], replacing the traditional dual 3 × 3 convolutions with two depthwise separable convolutions to reduce redundancy and improve computational efficiency. The overall architecture of YOLO11 is illustrated in Figure 4.

### 2.3. Improvements to the YOLO11

**Figure 5:**
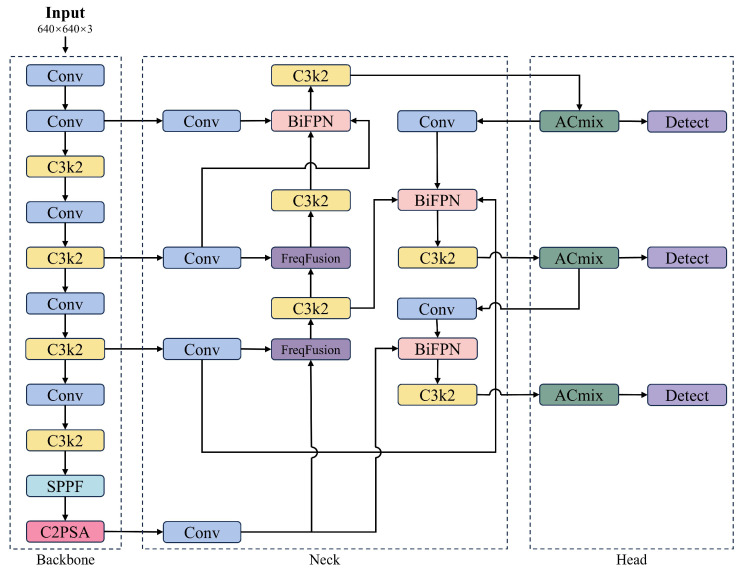
The network structure of AFBF-YOLO.

In this study, the AFBF-YOLO cherry tomato detection model is developed using YOLO11 as the base network. Firstly, the ACmix [19] self-attention mechanism was introduced to improve the recognition ability of cherry tomato features. Secondly, the proposed neck, FreqFusion-BiFPN, combines the frequency-aware filtering capabilities of FreqFusion [20] with the multi-scale fusion strengths of BiFPN [21] to improve the model’s ability to capture and express features of different ripeness levels in cherry tomatoes. Finally, Inner [22]-Focaler [23]-IoU was employed as the loss function in the improved model to enable more accurate evaluation of overlapping regions. The structure of AFBF-YOLO is shown in Figure 5.

#### 2.3.1. Attention Module

To enhance the model’s focus on fruit regions, suppress background clutter, and improve detection accuracy under occlusion, this study incorporates the ACmix attention mechanism. Compared to traditional attention mechanisms, ACmix is a hybrid model that integrates self-attention and convolutional operations, while maintaining low computational overhead compared to pure convolution or pure self-attention methods.

**Figure 6:**
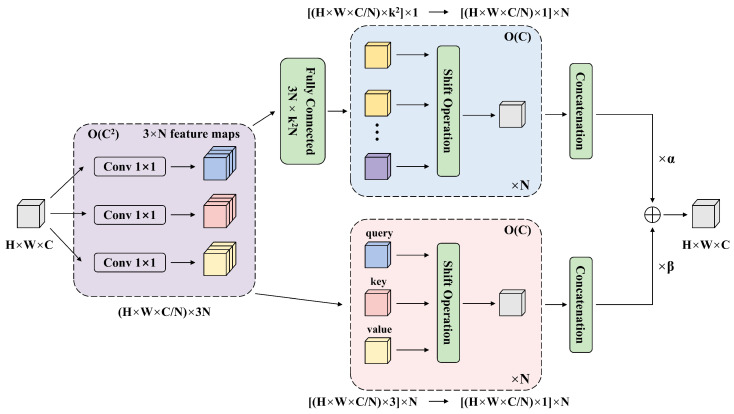
The structure of ACmix hybrid self-attention mechanism.

As shown in Figure 6, H, W, C, and N in the figure denote the length of the feature map, the width of the feature map, the number of channels, and the number of auto-attention heads, respectively. Firstly, the input features are projected by three 1 × 1 convolutions, from which three groups of feature maps of size 3 × N are obtained. Secondly, it is processed in two branches. In the self-attentive path, the intermediate features are assembled into N groups, each containing three 1 × 1 convolutional output features, and each corresponding sub-feature is used as a query, key, and value, respectively. In the convolutional path, the sub-features are processed through a $3N\times k^{2}N$ fully connected layer, where k denotes the size of the local receptive field. The processing includes shift, convolution and aggregation operations. Finally, the outputs of the two paths are summed up and their intensity can be controlled by two learnable scalars. This decomposition and reconstruction approach reduces redundant computations and improves computational efficiency while reducing the complexity of the model.

#### 2.3.2. Neck

The neck of YOLO11 retains the top-down path aggregation feature pyramid network (PAFPN), but it remains insufficient for capturing fine-grained features in cases of severe occlusion and overlapping cherry tomatoes. To address issues such as intra-class inconsistency and boundary misalignment in dense image prediction, a novel feature fusion module, FreqFusion-BiFPN, has been introduced through the integration of FreqFusion and BiFPN concepts.

Frequency-aware feature fusion (FreqFusion) incorporates an adaptive low-pass filter (ALPF), an offset generator, and an adaptive high-pass filter (AHPF). The ALPF attenuates high-frequency components within objects, reducing intra-category variation during upsampling. The offset generator enhances boundary clarity by resampling and substituting inconsistent features with more consistent ones. The AHPF restores high-frequency details lost during downsampling, improving boundary delineation. Overall, FreqFusion improves prediction accuracy by enhancing feature consistency and refining object boundaries.

**Figure 7:**
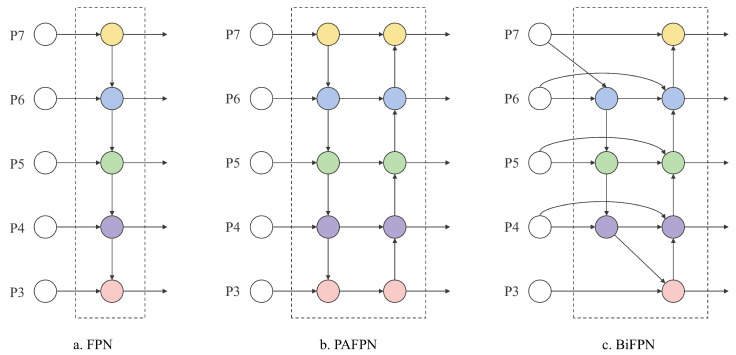
FPN, PAFPN, and BiFPN structures.

The bi-directional feature pyramid network (BiFPN) utilizes bidirectional cross-scale connectivity and weighted feature fusion to assign importance to features from different layers. It achieves efficient multi-source feature integration by removing low-contribution nodes and introducing lateral connections between input and output nodes at the same resolution. BiFPN treats bidirectional paths as processing units and optimizes fusion through iterative invocation, thereby reducing false positives caused by feature similarity. Figure 7 shows the comparison of different feature fusion methods.

We merge FreqFusion and BiFPN into FreqFusion-BiFPN to form a novel Neck network architecture, which combines the adaptive filtering and boundary optimization capabilities of FreqFusion with the efficient multi-scale feature fusion of BiFPN. Compared with the original BiFPN, FreqFusion-BiFPN introduces additional frequency-aware components, which refine feature fusion by enhancing intra-class consistency and boundary clarity across scales, while BiFPN employs scalar weights to balance multi-scale feature contributions, FreqFusion-BiFPN incorporates extra learnable parameters within these adaptive filters and offset modules, enabling more fine-grained control over spatial-frequency information. Although this design slightly increases model complexity, it also improves the robustness and precision of dense object detection in challenging scenarios such as occlusion and ripeness variation.

#### 2.3.3. Loss Function

The original YOLO11 model employs the CIoU loss function [29] to evaluate the overlap between predicted and ground-truth bounding boxes. However, CIoU primarily emphasizes overall box overlap while ignoring internal alignment, which limits localization accuracy for small targets and reduces robustness in complex or imbalanced scenarios. To address these issues, the Inner-Focaler-IoU loss function was proposed, integrating Inner-IoU and Focaler-IoU to measure the degree of overlap between two bounding boxes by incorporating the internal overlap region of the target, thereby enhancing detection performance for cherry tomatoes.

Focaler-IoU reconstructs the interior by linear interval mapping analyses and considers the impact of the distribution of difficult and simple samples in the bounding box regression on the regression results. When difficult samples such as hidden objects, small objects, or objects with similar backgrounds dominate, the relative weight of the difficult samples is increased to improve the detection performance. The mathematical expression of the Focaler-IoU is as shown in Equation (1):

(1) $$IoU^{focaler}=\left\{\begin{array}{cc} 0, & IoU<d\\ \frac{IoU-d}{u-d}, & d\leq IoU\leq u\\ 1, & IoU>u \end{array}\right.$$

The term $IoU^{focaler}$ represents the reconstructed Focaler-IoU, where IoU is the original IoU value, and *d* and *u* are both in the range of [0, 1]. By adjusting the values of *d* and *u*, Focaler-IoU can be directed to focus on different regression samples. After defining the Focaler-IoU, its corresponding loss can be defined as shown in Equation (2):

(2) $$L_{Focaler-IoU}=1-IoU^{focaler}$$

Compared with traditional IoU calculation methods, Inner-IoU not only considers the intersection region between the predicted and real frames but also further focuses on the ratio of the intersection to the predicted frames themselves, thus providing a more detailed and comprehensive evaluation perspective. Therefore, we optimize the model by introducing the Inner-IoU loss function, which can be integrated into existing IoU-based loss functions to improve the generalization capabilities of the model. The mathematical expression of the Inner-IoU is as shown in Equations (3)–(8):

(3) $$\left(b_{l}^{gt},b_{r}^{gt}\right)=\left(x_{c}^{gt}-\frac{w^{gt}\times \left(ratio\right)}{2},x_{c}^{gt}+\frac{w^{gt}\times \left(ratio\right)}{2}\right)$$

(4) $$\left(b_{t}^{gt},b_{b}^{gt}\right)=\left(y_{c}^{gt}-\frac{h^{gt}\times \left(ratio\right)}{2},y_{c}^{gt}+\frac{h^{gt}\times \left(ratio\right)}{2}\right)$$

(5) $$\left(b_{l},b_{r}\right)=\left(x_{c}-\frac{w\times (ratio)}{2},x_{c}+\frac{w\times (ratio)}{2}\right)$$

(6) $$\left(b_{t},b_{b}\right)=\left(y_{c}-\frac{h\times (ratio)}{2},y_{c}+\frac{h\times (ratio)}{2}\right)$$

(7) $$inter=\left[min\left(b_{r}^{gt},b_{r}\right)-max\left(b_{l}^{gt},b_{l}\right)\right]\times \left[min\left(b_{b}^{gt},b_{b}\right)-max\left(b_{t}^{gt},b_{t}\right)\right]$$

(8) $$union=\left(w^{gt}\times h^{gt}\right)\times {\left(ratio\right)}^{2}+\left(w\times h\right)\times {\left(ratio\right)}^{2}-inter$$

where $b^{gt}$ and *b* represent the ground truth (GT) box and the anchor box, respectively. $\left(x_{c}^{gt},y_{c}^{gt}\right)$ represents the center coordinates of the GT frame and auxiliary GT frame, and $\left(x_{c},y_{c}\right)$ represents the internal center points of the GT box. $w^{gt}$ and $h^{gt}$ denote the width and height of the GT frame, respectively, and *w* and *h* are the width and height of the anchor box. The variable ratio corresponds to the scale factor, which typically ranges from 0.5 to 1.5. The loss function incorporating these concepts is defined as shown in Equations (9) and (10).

(9) $$IoU^{inner}=\frac{inter}{union}$$

(10) $$L_{\mathrm{Inner}-\mathrm{IoU}}=1-{IoU}^{inner}=1-\frac{inter}{union}$$

**Figure 8:**
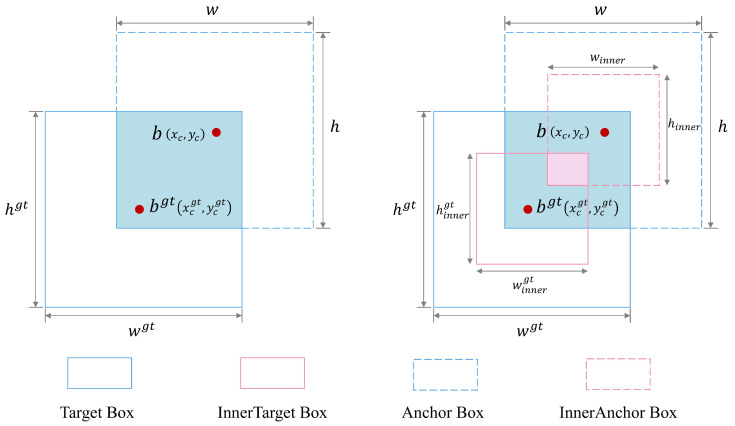
Description of Inner-Focaler-IoU.

The final composite IoU loss, termed Inner-Focaler-IoU, is defined as shown in Equations (11) and (12). As illustrated in Figure 8, the Inner-Focaler-IoU loss function emphasizes the intersection region. To better evaluate the quality of intersection regions, auxiliary bounding boxes have been introduced, whose linear sizes are related to the original boxes through the newly defined variable “ratio”.

(11) $${IoU}^{inner-focaler}=\left\{\begin{array}{cc} 0, & {IoU}^{inner}<d\\ \frac{{IoU}^{inner}-d}{u-d}, & d\leq {IoU}^{inner}\leq u\\ 1, & {IoU}^{inner}>u \end{array}\right.$$

(12) $$L_{\mathrm{Inner}-\mathrm{Focaler}-\mathrm{IoU}}=1-{IoU}^{inner-focaler}$$

### 2.4. Evaluation Metrics

This study employs five evaluation metrics for cherry tomato ripeness detection: precision (P), recall (R), F1 score, average precision (AP), and mean average precision (mAP). These metrics are defined as shown in Equations (13)–(17):

(13) $$P=\frac{TP}{TP+FP}$$

(14) $$R=\frac{TP}{TP+FN}$$

(15) $$F1=\frac{2\times P\times R}{P+R}$$

(16) $$AP=\int_{0}^{1}P\left(R\right)dR$$

(17) $$mAP=\frac{\sum_{i=1}^{n}AP_{i}}{n}$$

where TP (True Positives) refers to the number of correctly identified ripe tomatoes, while FP (False Positives) refers to cases where unripe tomatoes or other objects were incorrectly classified as ripe. FN (False Negatives) indicates the number of ripe tomatoes incorrectly classified as unripe or other categories. The mAP is calculated as the average of AP values across all categories, which comprehensively reflects the detection ability of the model on various categories of targets. In the formula, *n* is the total number of categories, and $AP_{i}$ is the average precision for the i-th category.

In addition to accuracy-related metrics, model efficiency was also assessed using the number of parameters, computational complexity, measured in Giga floating-point operations per second (GFLOPs), and inference time, to reflect hardware resource requirements.

## 3. Experiments and Results

### 3.1. Experimental Platform and Parameter Settings

This experiment is built on the PyTorch 2.1.0 deep learning framework and executed in an Anaconda environment. Table 2 shows the main experimental equipment environment configurations.

During training, the input image size of the network was set to 640 × 640 pixels. The training hyperparameter combinations are configured using the following specifications: the initial learning rate was set to 0.01, the momentum factor to 0.937, the weight decay to 0.0005, the batch size to 16, and the number of epochs to 200, with early stopping set at 100. To ensure the fairness of the experiments, no pretrained models were used during training. Furthermore, the stochastic gradient descent (SGD) algorithm was used as the optimization strategy.

### 3.2. Performance of AFBF-YOLO Network

**Figure 9:**
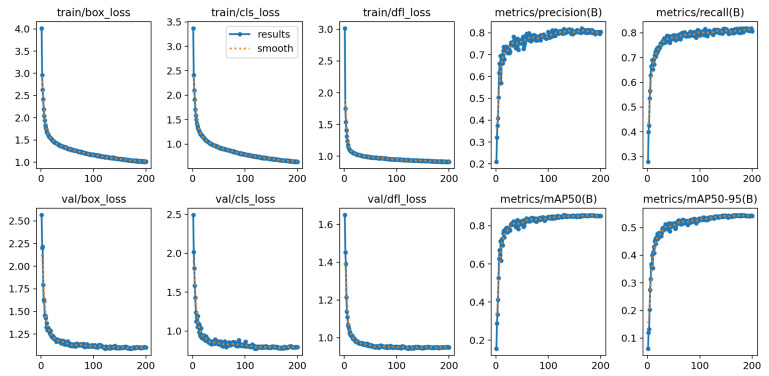
Experimental results of AFBF-YOLO model.

The performance of the AFBF-YOLO model was thoroughly evaluated after 200 training epochs. Figure 9 shows the training results of the AFBF-YOLO model. It is obvious from the figure that the precision (P), recall (R), mAP50, and mAP50-95 metrics show a clear trend of improvement as the number of iterations increases during the training process. All of these metrics reflect that our model successfully fitted the cherry tomato detection task.

**Figure 10:**
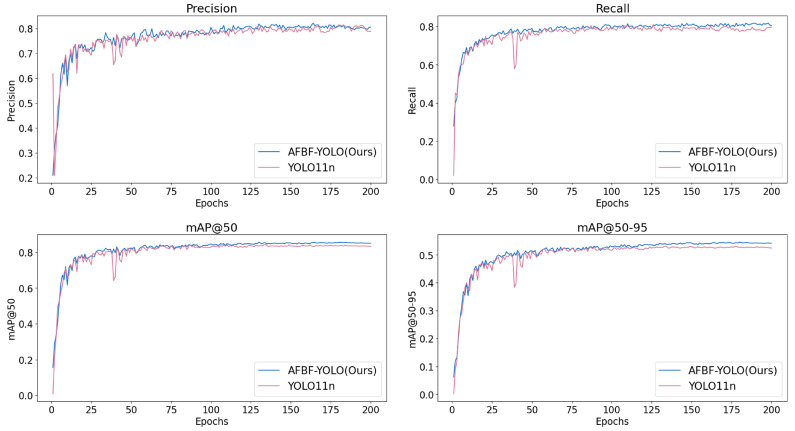
Comparison of indicators before and after model improvement.

Figure 10 further compares the performance of the original YOLO11 and the improved AFBF-YOLO model during training. With respect to the key metric mAP50, AFBF-YOLO demonstrates a more stable convergence trend and significantly reduced fluctuations in the precision and recall curves. After 200 epochs, the improved model outperforms the baseline YOLO11 in multiple evaluation metrics, indicating enhanced detection accuracy and better generalization capability.

**Figure 11:**
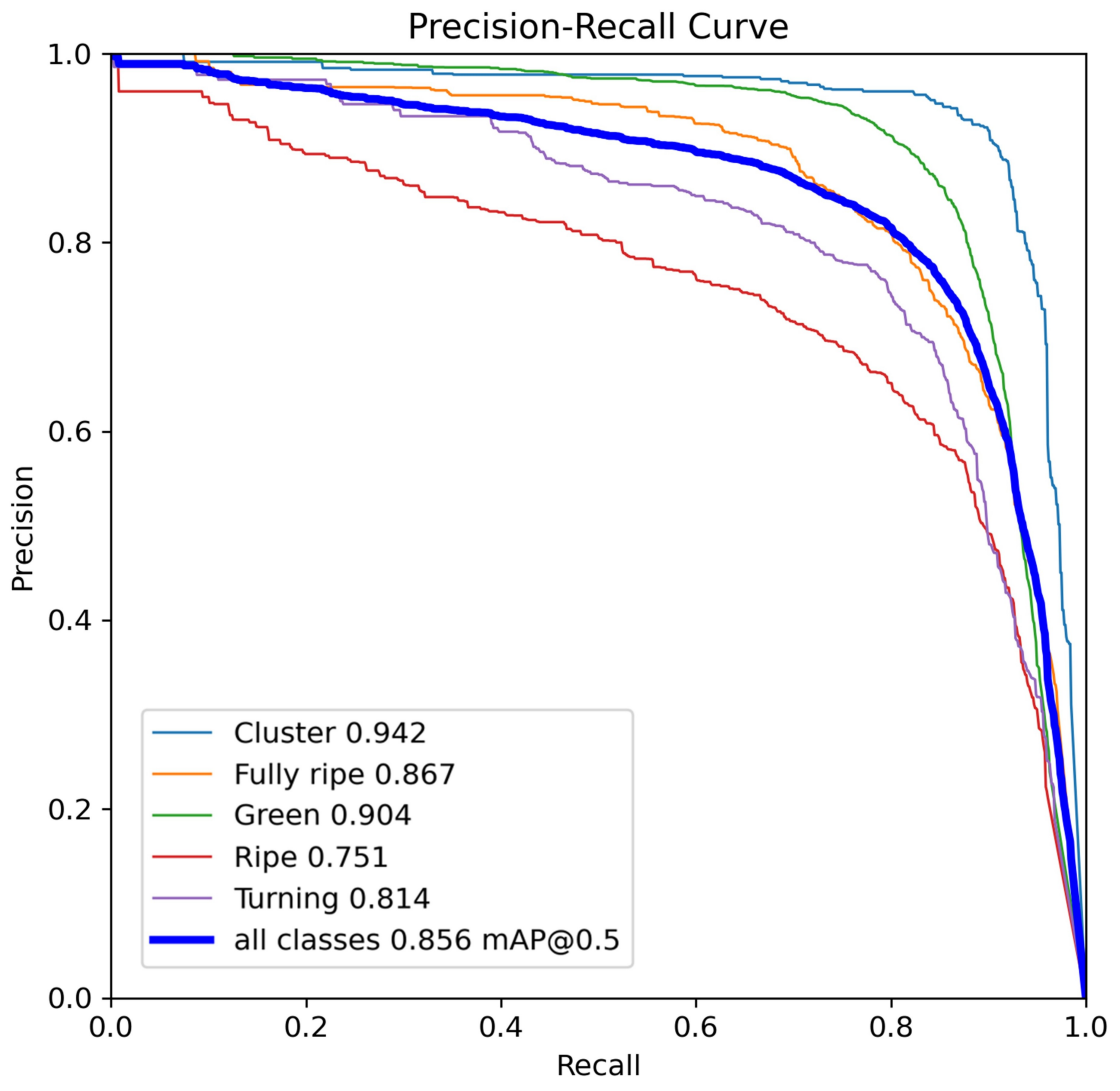
Precision–Recall curve of AFBF-YOLO model.

To further evaluate the model’s ability to detect cherry tomato maturity across categories, Figure 11 shows the precision–recall (P-R) curves of the model for the different classes of objects (“cluster”, “fully ripe”, “ripe”, “turning”, and “green”). The P-R curve of all classes is 0.856 for mAP@0.5, but there is some variation in performance across categories. The model achieves higher accuracy for the green and fully ripe classes, while performance is relatively lower for the turning and ripe stages.

**Figure 12:**
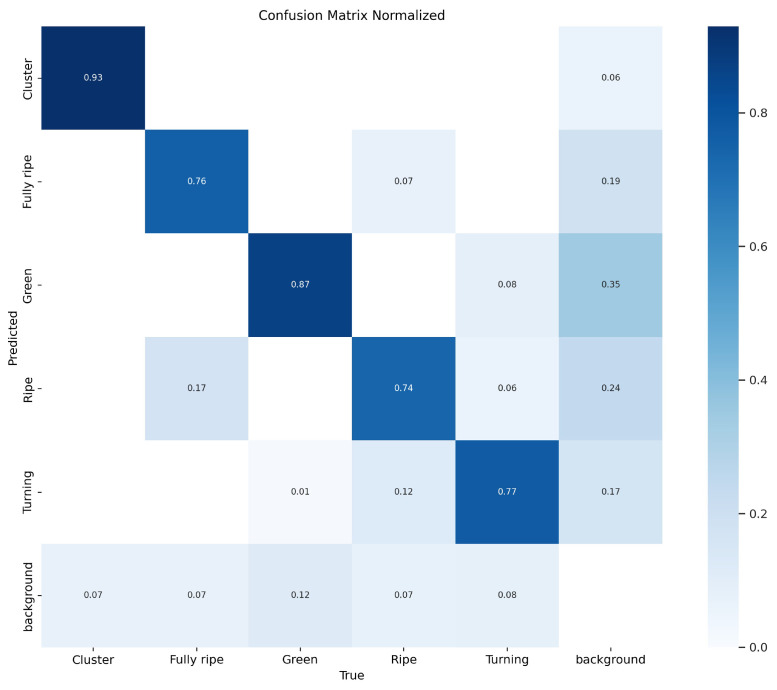
Normalized confusion matrix of AFBF-YOLO model.

In addition, Figure 12 shows the normalized confusion matrix, which evaluates the classification performance of the model in different categories. This normalization allows us to visually compare classification accuracies between categories and identify the categories in which the model performs better or worse. The color intensity of each cell indicates predictive accuracy, and darker shading indicates higher accuracy. The model’s accuracy for detecting cluster, fully ripeness, green ripeness, ripeness, and turning ripeness was 0.93, 0.76, 0.87, 0.74, and 0.77, respectively. The relatively lower accuracy for the turning and ripe classes can be attributed to misclassification: around 12% of turning tomatoes were predicted as ripe, and approximately 17% of ripe tomatoes were mislabeled as fully ripe.

### 3.3. Visual Analysis

**Figure 13:**
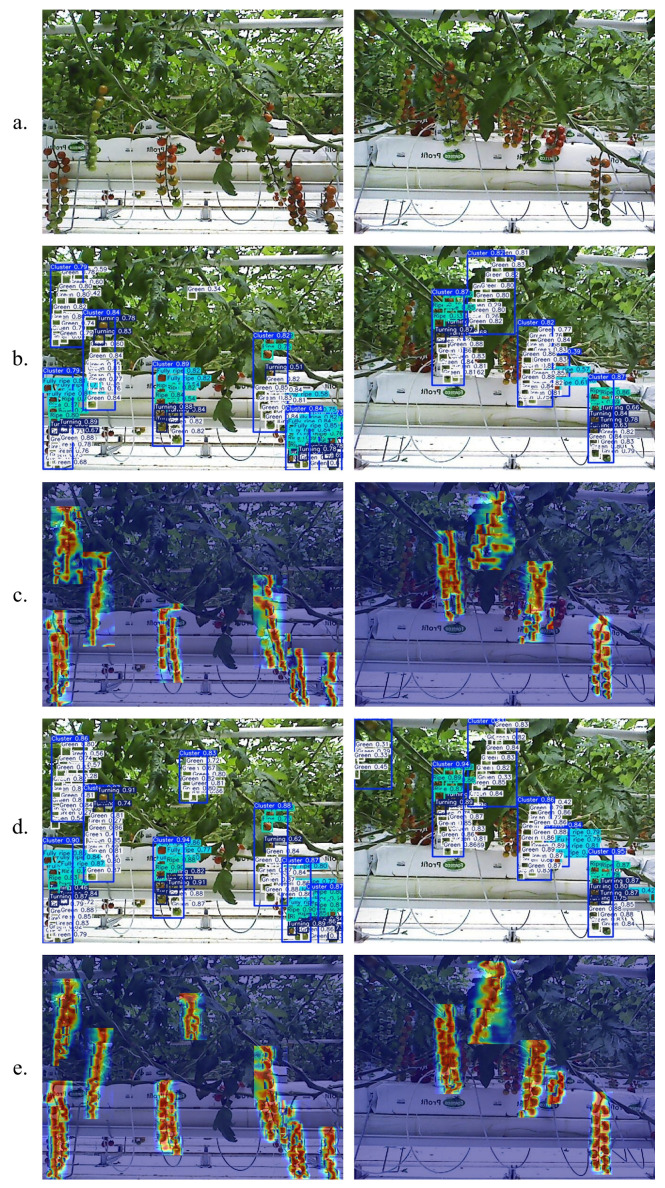
Comparison of heatmaps of YOLO11 model and AFBF-YOLO model. (**a**) Original images. (**b**) The detection results of YOLO11n. (**c**) The detection heatmaps of YOLO11n. (**d**) The detection results of AFBF-YOLO. (**e**) The detection heatmaps of AFBF-YOLO.

To visually demonstrate the performance of cherry tomato ripeness detection in greenhouse environments, Figure 13 presents heatmap visualizations generated using gradient-weighted class activation mapping (Grad-CAM [30]) for both YOLO11n and AFBF-YOLO, highlighting the key regions attended by each model. The heatmaps illustrate object activation intensity through color variations: warmer tones (e.g., red and yellow) indicate regions contributing more to maturity prediction, while cooler tones (e.g., blue and green) represent regions with lower contribution.

During the detection process, we set the confidence threshold to 0.5 and the IoU threshold to 0.65. As shown in Figure 13b,c, the use of YOLO11 to detect cherry tomato maturity in natural environments revealed that the distribution of high-attention areas was relatively sparse and some cherry tomatoes were not effectively labeled. In contrast, the heatmap from the AFBF-YOLO model shows denser and more continuous areas of high interest, highlighting clusters of highly activated regions. These visual results confirm that the optimized model exhibits stronger attention focusing on relevant cherry tomato regions under complex background conditions.

### 3.4. Ablation Experiments

To evaluate the individual contributions of each proposed module, a series of ablation experiments were conducted. The original YOLO11 model was used as the baseline, with each of the ACmix, FreqFusion-BiFPN, and Inner-Focaler-IoU modules added incrementally to assess their effect on model performance. These experiments were designed to isolate and measure the performance impact of each architectural enhancement: A (Addition of attention mechanism), B (Improvement of the neck), and C (Optimization of the loss function).

As indicated in Table 3, the introduction of the ACmix, FreqFusion-BiFPN, and Inner-Focaler-IoU modules improved the detection performance of cherry tomatoes of all ripeness levels in complex environments to different degrees. Specifically, after adding ACmix, precision increases from 80.5% to 81.8%, recall from 79.7% to 80.3%, F1 score from 80.1% to 81.0%, mAP50 from 83.8% to 84.4%, and mAP50-95 from 53.1% to 53.3%, indicating that ACmix enhances the model’s ability to detect key features effectively. When the FreqFusion-BiFPN module is applied independently, the model achieves an mAP50 of 84.5% and an mAP50-95 of 53.4%, resulting in improvements of 0.7% and 1.1%, respectively. This improvement is likely due to the ability of FreqFusion-BiFPN to integrate features across multiple scales, allowing the model to better handle diverse inputs and boost detection performance. The model with Inner-Focaler-IoU alone sees an increase of 1.1% and 1.6% in precision and recall, respectively, while the computational complexity does not increase significantly.

The detection performance of the model is further improved when the modules are applied in combination. When both the ACmix module and Inner-Focaler-IoU are employed, the performance of the model improves significantly on all evaluation metrics. Specifically, the F1 score increased to 80.8%, and mAP50 and mAP50-90 reached 84.9% and 53.6%, respectively. These results suggest that the ACmix mechanism and Inner-Focaler-IoU can effectively complement each other: the former suppresses non-targeted information, while the latter excels at extracting key features, thus improving feature representation. In contrast, combining the ACmix and FreqFusion-BiFPN modules results in a slight decrease in other performance metrics, although mAP50 and mAP50-95 improved to 84.7% and 53.9%, respectively. This may be attributed to potential interference between attention-based and frequency-based feature representations, as well as optimization trade-offs introduced by concurrent module interactions. Future work could explore adaptive fusion strategies or dynamic weighting mechanisms to better harmonize these modules.

Finally, by combining ACmix, FreqFusion-BiFPN, and Inner-Focaler-IoU, the model demonstrates optimal overall performance. AFBF-YOLO achieves a precision of 81.2%, recall of 81.3%, mAP50 of 85.6%, and mAP50-95 of 54.3%. Furthermore, the addition of multiple modules introduces only a slight increase in computational complexity (GFLOPs), while yielding substantial performance gains. These results confirm that the improved model can effectively recognize cherry tomatoes in complex environments while balancing performance and efficiency.

### 3.5. Performance Comparison with YOLO Series Model

To further validate the effectiveness of the improved network model in this paper in the cherry tomato detection task in the natural environment, it is compared with the experimental results of other mainstream target detection models at this stage, namely, YOLOv5n [31], YOLOv8n, YOLOv9t [32], YOLOv10n, and YOLO11n. The above method is trained using the same dataset and device, and then the object detection models are compared using the same test set to compare the object detection models. The specific results of the comparison experiments are shown in Table 4.

As shown in Table 4, the AFBF-YOLO model shows excellent performance compared to the other models. The AFBF-YOLO model achieved 81.2% precision (P), which is higher than all models except YOLOv5n and YOLOv8n. For recall (R), it achieved 81.3%, which also shows good performance in this metric. In addition, the F1 score of the AFBF-YOLO model is significantly better than the other comparative models. This indicates that the model not only accurately identifies targets but also minimizes target misses. In terms of mAP50, AFBF-YOLO achieved 85.6%, outperforming all models in this regard, implying its better detection at high confidence. Taking into account mAP50-95, AFBF-YOLO achieved 54.3%, outperforming all other models, indicating that it has the best robustness under different IoU thresholds. However, the computational complexity of AFBF-YOLO is 7.4 GFLOPs, and the average inference time is 8.1 ms, slightly higher than YOLOv5n and YOLO11n, due to the added attention, feature fusion, and loss function modules. Nevertheless, this moderate increase is acceptable given the notable gains in precision and stability [33], with the latency remaining within the acceptable range for real-time deployment on edge devices [34]. Overall, AFBF-YOLO achieves high precision and recall while maintaining reasonable computational efficiency, making it suitable for practical applications requiring both reliability and responsiveness in complex greenhouse environments.

## 4. Discussion

### 4.1. Discussion of the Current Research Status

The rapid advancement of deep learning has spurred the proliferation of sophisticated network architectures [35,36], which offer promising solutions for tomato maturity detection. For example, Zeng et al. [37] improved the YOLOv5 model to achieve real-time localization and maturity classification of tomato fruits on low computing power platforms. Wang et al. [38] proposed an improved Faster R-CNN model with an average precision of 94.16%. Huang et al. [39] developed a multi-scale AITP-YOLO model for maturity detection, achieving an mAP50 of 92.6%. Wei et al. [40] proposed a lightweight tomato ripeness detection model, GFS-YOLO11, achieving a precision of 92%.

Most existing detection models focus primarily on the ripeness of tomato fruits, and the above methods do not simultaneously perform tomato bunch identification and fruit ripeness detection. The AFBF-YOLO model proposed in this study not only performs well in bunch and fruit detection but also effectively classifies four ripeness levels under greenhouse conditions, while the proposed model demonstrates superior overall performance compared with YOLO series baselines, it is noted that the detection accuracy is slightly lower than that reported in some prior works focused solely on fruit or cluster detection. This is to be expected given that AFBF-YOLO is designed to perform both per-fruit localization and ripeness classification simultaneously, which is a more complex task than single-object or cluster-level detection. The model must identify bunches and individual tomatoes under occlusion, variable lighting, and overlapping growth, while also distinguishing subtle differences in maturity stages. In contrast, previous studies such as Chai et al. [41] and Liang et al. [42] primarily targeted either cluster detection or ripeness classification alone, which offer valuable insights under their respective task definitions. Therefore, despite marginally lower precision in certain metrics, our model achieves more comprehensive detection functionality.

The improvement is mainly attributed to integrating three novel modules: the ACmix hybrid attention mechanism [19], the FreqFusion [20]-BiFPN [21] neck structure, and the Inner [22]-Focaler [23]-IoU loss function. The ACmix module enhances the model’s ability to suppress background interference and focus on critical fruit regions, especially under occlusion or uneven lighting. The FreqFusion-BiFPN module significantly improves multi-scale feature fusion, making the model more robust to variation in fruit size and maturity levels. Furthermore, the Inner-Focaler-IoU loss addresses the limitations of traditional IoU-based metrics by emphasizing the overlap quality in the inner bounding box region, thereby improving localization performance.

Compared with previous studies that focused solely on YOLO backbone improvements or data augmentation strategies, this work provides a more holistic approach by simultaneously enhancing attention, feature fusion, and loss computation. The ablation experiments confirm that each proposed component independently contributes to performance gains, and the combination yields the best results with only a modest increase in parameters and computational complexity.

### 4.2. Limitations and Future Work

Despite the notable improvements, several limitations remain. Firstly, The dataset used for training and evaluation was collected from a single greenhouse on a single day, which may limit the model’s generalizability across different locations, seasons, lighting conditions, or tomato cultivars. This lack of variability introduces challenges related to environmental diversity and temporal robustness. Expanding the dataset to include a wider range of growing conditions is essential for improving cross-domain performance. Additionally, as shown in Table 1, the dataset exhibits a notable class imbalance, particularly between the “green” class (61,694 samples) and the “cluster” class (13,149 samples). Such an imbalance may lead the model to favor dominant classes and affect the detection accuracy of underrepresented categories. Although no class-balancing techniques (e.g., resampling or reweighting) were applied [43], several measures were taken to mitigate these effects. These include extensive data augmentation and the use of the Inner-Focaler-IoU loss function that emphasizes difficult samples. Nonetheless, we acknowledge that the imbalance may still influence detection performance, particularly for classes with fewer instances. Finally, we acknowledge that our current research concentrates on algorithmic-level model design and performance validation, without testing hardware deployment in real agricultural scenarios. This leaves edge device compatibility, like actual deployment latency, unexplored.

The model also shows confusion between the “turning” and “ripe” stages, highlighting the challenge of distinguishing transitional maturity levels based on limited visual cues. A detailed statistical analysis of misclassifications and missed detections across ripeness categories reveals several key factors contributing to reduced recognition accuracy. The main contributing factors are summarized as follows:

(1) The “turning” and “ripe” classes exhibit gradual color transitions and overlapping features in hue and texture, especially when ripeness gradients are ambiguous under natural lighting [44,45].
(2) Dense fruit clustering and interference from leaves or stems often cause partial or complete occlusion, leading to incomplete feature representation and increased false negatives and localization errors.
(3) Inconsistent illumination and shadowing introduce variability in color and texture, distorting the visual appearance of fruit surfaces, especially under partial shading or backlighting.
(4) In the early “green” stage, fruit color and texture often blend with the surrounding vegetation. The small size and low signal-to-background contrast impair fruit contour discrimination, leading to missed detections.
(5) Labeling noise and human subjectivity during manual annotation may contribute to boundary inconsistencies and marginal misclassifications.

In subsequent research, we intend to expand the dataset with samples from different geographic regions, seasons, and tomato varieties, particularly by increasing the number of images capturing the color transition and mature stages of tomatoes, thereby further improving precision agriculture technology. Moreover, detection precision and model generalization can be further enhanced by modifying the detection models or incorporating spectral imaging technologies. We hope to extend the AFBF-YOLO network to more tomato varieties and explore its application to ripeness detection of other fruits in clusters. We will also conduct performance evaluation across diverse hardware platforms to validate its suitability for real-time robotic harvesting, employing filter pruning methods [46] and distillation techniques [47] to further reduce inference latency.

## 5. Conclusions

In complex greenhouse environments with shading, overlapping fruits, and subtle color differences, accurate detection of cherry tomato clusters and ripeness stages is critical for intelligent harvesting systems. This study proposes the AFBF-YOLO multi-task detection model, which incorporates the ACmix attention mechanism, FreqFusion-BiFPN, and Inner-Focaler-IoU loss function to achieve bunch identification and ripeness detection of cherry tomato fruits. Experimental results on the cherry tomato dataset, which contains five data enhancement methods, show that our proposed method achieves significant performance improvements. The precision, recall, F1 score, mAP50, and mAP50-95 of the model reach 81.2%, 81.3%, 81.2%, 85.6%, and 54.3%, respectively. The parameters, FLOPS and inference time are 2.73 M, 7.4 G, and 8.1 ms, respectively. The ablation experiments further validate the effectiveness of the individual modules and demonstrate that the combination of ACmix, FreqFusion-BiFPN, and Inner-Focaler-IoU can synergistically improve the overall performance of the model. In summary, AFBF-YOLO is suitable for accurate target localization and ripeness grading in automatic cherry tomato picking, which provides a valuable reference for intelligent fruit picking in agriculture.

## Figures and Tables

**Table 1:** Number of samples for each class in the training and test sets.

Class	Training Set	Test Set	Total
Cluster	12,624	525	13,149
Fully ripe	29,340	1217	30,557
Green	59,032	2612	61,694
Ripe	28,356	1031	29,387
Turning	19,842	796	20,638

**Table 2:** Experimental environment configuration.

Environment Configuration	Parameter
Operating system	Windows 11
CPU	Intel(R) Xeon(R) Gold 8481
GPU	NVIDIA RTX 4090 16 GB
Development environment	Visual Studio Code 1.98
Programming language	Python 3.10.8
Deep learning framework	Pytorch 2.1.0
Operating platform	Cuda 12.1

**Table 3:** Results of ablation experiments. (‘✓’ indicates the introduction of the mechanism, while ‘×’ indicates its absence.)

A	B	C	P (%)	R (%)	F1 Score (%)	${\mathrm{mAP}}_{50}$ (%)	${\mathrm{mAP}}_{50-95}$ (%)	Parameters (M)	FLOPS (G)
×	×	×	80.5	79.7	80.1	83.8	53.1	2,590,815	6.4
✓	×	×	81.8	80.3	81.0	84.4	53.3	2,887,161	7.1
×	✓	×	81.5	80.8	81.1	84.5	53.4	2,755,683	8.1
×	×	✓	81.6	81.3	81.4	84.3	53.3	2,590,815	6.4
✓	✓	×	80.4	79.7	80.0	84.7	53.9	2,730,749	7.4
✓	×	✓	81.4	80.3	80.8	84.9	53.6	2,879,473	7.0
×	✓	✓	79.9	80.9	80.4	84.4	53.9	2,434,403	6.8
✓	✓	✓	81.2	81.3	81.2	85.6	54.3	2,730,749	7.4

**Table 4:** Comparison results of YOLO algorithms.

Model	P (%)	R (%)	F1 Score (%)	${\mathrm{mAP}}_{50}$ (%)	${\mathrm{mAP}}_{50-95}$ (%)	Parameters (M)	FLOPS (G)	Time (ms)
YOLOv5n	81.2	79.7	80.4	83.8	52.7	2,503,919	7.1	3.3
YOLOv8n	81.6	80.2	80.9	83.9	52.7	3,006,623	8.1	5.8
YOLOv9t	80.7	80.4	80.5	84.7	53.8	2,006,383	7.9	6.8
YOLOv10n	79.8	78.0	78.9	84.0	52.8	2,696,366	8.2	6.2
YOLO11n	80.5	79.7	80.1	83.8	53.1	2,590,815	6.4	3.7
AFBF-YOLO (Ours)	81.2	81.3	81.2	85.6	54.3	2,730,749	7.4	8.1

## Data Availability

Data available on request from the authors.

## References

[1] Collins E.J., Bowyer C., Tsouza A., Chopra M. (2022). Tomatoes: An extensive review of the associated health impacts of tomatoes and factors that can affect their cultivation. Biology.

[2] Raffo A., Leonardi C., Fogliano V., Ambrosino P., Salucci M., Gennaro L., Bugianesi R., Giuffrida F., Quaglia G. (2002). Nutritional value of cherry tomatoes (Lycopersicon esculentum cv. Naomi F1) harvested at different ripening stages. J. Agric. Food Chem..

[3] Feng Q., Zou W., Fan P., Zhang C., Wang X. (2018). Design and test of robotic harvesting system for cherry tomato. Int. J. Agric. Biol. Eng..

[4] Jin T., Han X. (2024). Robotic arms in precision agriculture: A comprehensive review of the technologies, applications, challenges, and future prospects. Comput. Electron. Agric..

[5] Ampatzidis Y., Partel V., Costa L. (2020). Agroview: Cloud-based application to process, analyze and visualize UAV-collected data for precision agriculture applications utilizing artificial intelligence. Comput. Electron. Agric..

[6] Li M., Wu F., Wang F., Zou T., Li M., Xiao X. (2024). CNN-MLP-Based configurable robotic arm for Smart Agriculture. Agriculture.

[7] Saleem M.H., Potgieter J., Arif K.M. (2021). Automation in agriculture by machine and deep learning techniques: A review of recent developments. Precis. Agric..

[8] Xiao F., Wang H., Xu Y., Zhang R. (2023). Fruit detection and recognition based on deep learning for automatic harvesting: An overview and review. Agronomy.

[9] Hou G., Niu R., Chen H. (2025). A visual recognition method for the growth posture of small tomatoes under a complex greenhouse background. Comput. Electron. Agric..

[10] Rizzo M., Marcuzzo M., Zangari A., Gasparetto A., Albarelli A. (2023). Fruit ripeness classification: A survey. Artif. Intell. Agric..

[11] Yoshida T., Fukao T., Hasegawa T. (2020). Cutting point detection using a robot with point clouds for tomato harvesting. J. Robot. Mechatronics.

[12] Meng Z., Du X., Xia J., Ma Z., Zhang T. (2024). Real-time statistical algorithm for cherry tomatoes with different ripeness based on depth information mapping. Comput. Electron. Agric..

[13] Tenorio G.L., Caarls W. (2021). Automatic visual estimation of tomato cluster maturity in plant rows. Mach. Vis. Appl..

[14] Dai G., Hu L., Wang P., Rong J. (2022). Tracking and Counting Method for Tomato Fruits Scouting Robot in Greenhouse. Proceedings of the International Conference on Intelligent Robotics and Applications.

[15] Rong J., Zhou H., Zhang F., Yuan T., Wang P. (2023). Tomato cluster detection and counting using improved YOLOv5 based on RGB-D fusion. Comput. Electron. Agric..

[16] Li Y., Feng Q., Liu C., Xiong Z., Sun Y., Xie F., Li T., Zhao C. (2023). MTA-YOLACT: Multitask-aware network on fruit bunch identification for cherry tomato robotic harvesting. Eur. J. Agron..

[17] Li X., Ma N., Han Y., Yang S., Zheng S. AHPPEBot: Autonomous robot for tomato harvesting based on phenotyping and pose estimation. Proceedings of the 2024 IEEE International Conference on Robotics and Automation (ICRA).

[18] Chen W., Liu M., Zhao C., Li X., Wang Y. (2024). MTD-YOLO: Multi-task deep convolutional neural network for cherry tomato fruit bunch maturity detection. Comput. Electron. Agric..

[19] Pan X., Ge C., Lu R., Song S., Chen G., Huang Z., Huang G. On the integration of self-attention and convolution. Proceedings of the IEEE/CVF Conference on Computer Vision and Pattern Recognition.

[20] Chen L., Fu Y., Gu L., Yan C., Harada T., Huang G. (2024). Frequency-aware feature fusion for dense image prediction. IEEE Trans. Pattern Anal. Mach. Intell..

[21] Tan M., Pang R., Le Q.V. EfficientDet: Scalable and efficient object detection. Proceedings of the IEEE/CVF Conference on Computer Vision and Pattern Recognition.

[22] Zhang H., Xu C., Zhang S. (2023). Inner-IoU: More effective intersection over union loss with auxiliary bounding box. arXiv.

[23] Zhang H., Zhang S. (2024). Focaler-IoU: More focused intersection over union loss. arXiv.

[24] Li X., Chen W., Wang Y., Yang S., Wu H., Zhao C. (2023). Design and experiment of an automatic cherry tomato harvesting system based on cascade vision detection. Trans. Chin. Soc. Agric. Eng..

[25] Wang Z., Wang R., Wang M., Lai T., Zhang M. (2024). Self-supervised transformer-based pre-training method with General Plant Infection dataset. Proceedings of the Chinese Conference on Pattern Recognition and Computer Vision (PRCV).

[26] Khanam R., Hussain M. (2024). Yolov11: An overview of the key architectural enhancements. arXiv.

[27] Varghese R., Sambath M. Yolov8: A novel object detection algorithm with enhanced performance and robustness. Proceedings of the 2024 International Conference on Advances in Data Engineering and Intelligent Computing Systems (ADICS).

[28] Wang A., Chen H., Liu L., Chen K., Lin Z., Han J., Ding G. (2024). Yolov10: Real-time end-to-end object detection. Adv. Neural Inf. Process. Syst..

[29] Zheng Z., Wang P., Ren D., Liu W., Ye R., Hu Q., Zuo W. (2021). Enhancing geometric factors in model learning and inference for object detection and instance segmentation. IEEE Trans. Cybern..

[30] Selvaraju R.R., Cogswell M., Das A., Vedantam R., Parikh D., Batra D. Grad-CAM: Visual explanations from deep networks via gradient-based localization. Proceedings of the IEEE International Conference on Computer Vision.

[31] Jocher G., Stoken A., Borovec J., Changyu L., Hogan A., Diaconu L., Poznanski J., Yu L., Rai P., Ferriday R. (2020). Ultralytics/Yolov5: V3.0; Zenodo. https://zenodo.org/records/3983579.

[32] Wang C.Y., Yeh I.H., Mark Liao H.Y. (2024). Yolov9: Learning what you want to learn using programmable gradient information. Proceedings of the European Conference on Computer Vision.

[33] Tan M., Le Q.V. Rethinking model scaling for convolutional neural networks. Proceedings of the International Conference on Machine Learning.

[34] Qiu Z., Wang F., Li T., Liu C., Jin X., Qing S., Shi Y., Wu Y., Liu C. (2025). LGWheatNet: A Lightweight Wheat Spike Detection Model Based on Multi-Scale Information Fusion. Plants.

[35] Qin Y.M., Tu Y.H., Li T., Ni Y., Wang R.F., Wang H. (2025). Deep Learning for Sustainable Agriculture: A Systematic Review on Applications in Lettuce Cultivation. Sustainability.

[36] Wang R.F., Su W.H. (2024). The application of deep learning in the whole potato production Chain: A Comprehensive review. Agriculture.

[37] Zeng T., Li S., Song Q., Zhong F., Wei X. (2023). Lightweight tomato real-time detection method based on improved YOLO and mobile deployment. Comput. Electron. Agric..

[38] Wang Z., Ling Y., Wang X., Meng D., Nie L., An G., Wang X. (2022). An improved Faster R-CNN model for multi-object tomato maturity detection in complex scenarios. Ecol. Inform..

[39] Huang W., Liao Y., Wang P., Chen Z., Yang Z., Xu L., Mu J. (2025). AITP-YOLO: Improved tomato ripeness detection model based on multiple strategies. Front. Plant Sci..

[40] Wei J., Ni L., Luo L., Chen M., You M., Sun Y., Hu T. (2024). GFS-YOLO11: A Maturity Detection Model for Multi-Variety Tomato. Agronomy.

[41] Chai S., Wen M., Li P., Zeng Z., Tian Y. (2025). DCFA-YOLO: A Dual-Channel Cross-Feature-Fusion Attention YOLO Network for Cherry Tomato Bunch Detection. Agriculture.

[42] Liang X., Jia H., Wang H., Zhang L., Li D., Wei Z., You H., Wan X., Li R., Li W. (2025). ASE-YOLOv8n: A Method for Cherry Tomato Ripening Detection. Agronomy.

[43] Obuchi T., Tanaka T. (2024). When resampling/reweighting improves feature learning in imbalanced classification?: A toy-model study. arXiv.

[44] Xu Y., Liu S. (2024). Botanical-based simulation of color change in fruit ripening: Taking tomato as an example. Comput. Animat. Virtual Worlds.

[45] Ropelewska E., Szwejda-Grzybowska J. (2022). Relationship of Textures from Tomato Fruit Images Acquired Using a Digital Camera and Lycopene Content Determined by High-Performance Liquid Chromatography. Agriculture.

[46] Jeon J., Kang J.K., Kim Y. Filter pruning method for inference time acceleration based on YOLOX in edge device. Proceedings of the 2022 19th International SoC Design Conference (ISOCC).

[47] Gou J., Yu B., Maybank S.J., Tao D. (2021). Knowledge distillation: A survey. Int. J. Comput. Vis..

